# Changes in cerebral oxygenation during hemodialysis before and after carotid artery stenting

**DOI:** 10.1016/j.radcr.2022.04.054

**Published:** 2022-05-27

**Authors:** Hisashi Sato, Susumu Ookawara, Kiyonori Ito, Yuichiro Ueda, Keiji Hirai, Yoshikazu Yoshino, Yoshiyuki Morishita

**Affiliations:** a Division of General Medicine, First Department of Integrated Medicine, Saitama Medical Center, Jichi Medical University, Saitama, Japan; b Division of Nephrology, First Department of Integrated Medicine, Saitama Medical Center, Jichi Medical University, Saitama, Japan; c Department of Endovascular Surgery, Saitama Medical Center, Jichi Medical University, Saitama, Japan

**Keywords:** Carotid artery stenosis, Carotid artery stenting, Cerebral oxygenation, Hemodialysis, Regional oxygen saturation

## Abstract

A 68-year-old man received hemodialysis (HD) for the treatment of end-stage renal failure for 6 years. Five years prior to carotid artery stenting (CAS), a neck ultrasound performed to screen for carotid atherosclerosis revealed an asymptomatic right internal carotid artery stenosis. One month prior, the stenotic lesion progressed to 74% by cerebral angiography; therefore, CAS was performed. To evaluate the influence of right internal carotid artery stenosis on the intradialytic cerebral circulation and oxygenation, cerebral regional oxygen saturation (rSO_2_) at bilateral forehead was measured using the INVOS 5100c oxygen saturation monitor (Covidien Japan, Japan) during HD before and after CAS. Before CAS, right cerebral rSO_2_ was maintained during HD, whereas left cerebral rSO_2_ gradually increased from the initiation to end of HD. However, the differences of intradialytic cerebral rSO_2_ changes between bilateral sides disappeared after CAS. In the present case, before CAS, the intradialytic increase in left cerebral rSO_2_ might reflect the increase in the left cerebral blood flow to compensate for the ultrafiltration-associated decreases in the right cerebral blood flow and perfusion pressure. Furthermore, the preserved right cerebral rSO_2_ before CAS might reflect the mechanism maintaining the right cerebral blood flow from the collateralized circle of Willis during HD. Throughout our experience, cerebral oxygenation monitoring during HD might disclose intradialytic changes in cerebral blood flow distribution between the ipsilateral and contralateral side in HD patients with carotid artery stenosis.

## Introduction

Cerebrovascular disease is the fourth leading cause of death, which occupies 6.0%, and its prevention is critical in the field of dialysis therapy in Japan [1]. Furthermore, in patients undergoing hemodialysis (HD), diabetes mellitus and hypertension resulting in chronic kidney disease (CKD) and CKD-mineral and bone metabolism disorders are frequently associated with accelerated atherosclerosis and could lead to the high prevalence of carotid artery stenosis [2], [3], [4]. Carotid artery stenting (CAS) is considered feasible and effective for the prevention of cerebrovascular disease because of the recent favorable findings of carotid revascularization, including CAS, for the dialysis patients with carotid artery stenosis [5,6].

Near-infrared spectroscopy (NIRS) has been used to measure the regional oxygen saturation (rSO_2_), which is a tissue oxygenation marker. Continuous and non-invasive monitoring of cerebral rSO_2_ was reported in the clinical setting of HD therapy [7,8]. However, to date, no studies have reported the association between the CAS procedure and changes in cerebral oxygenation during HD. We herein focused on changes in cerebral oxygenation during HD before and after CAS and evaluated the effect of CAS on cerebral oxygenation in an HD patient with asymptomatic carotid artery stenosis.

## Case presentation

**Fig. 1:**
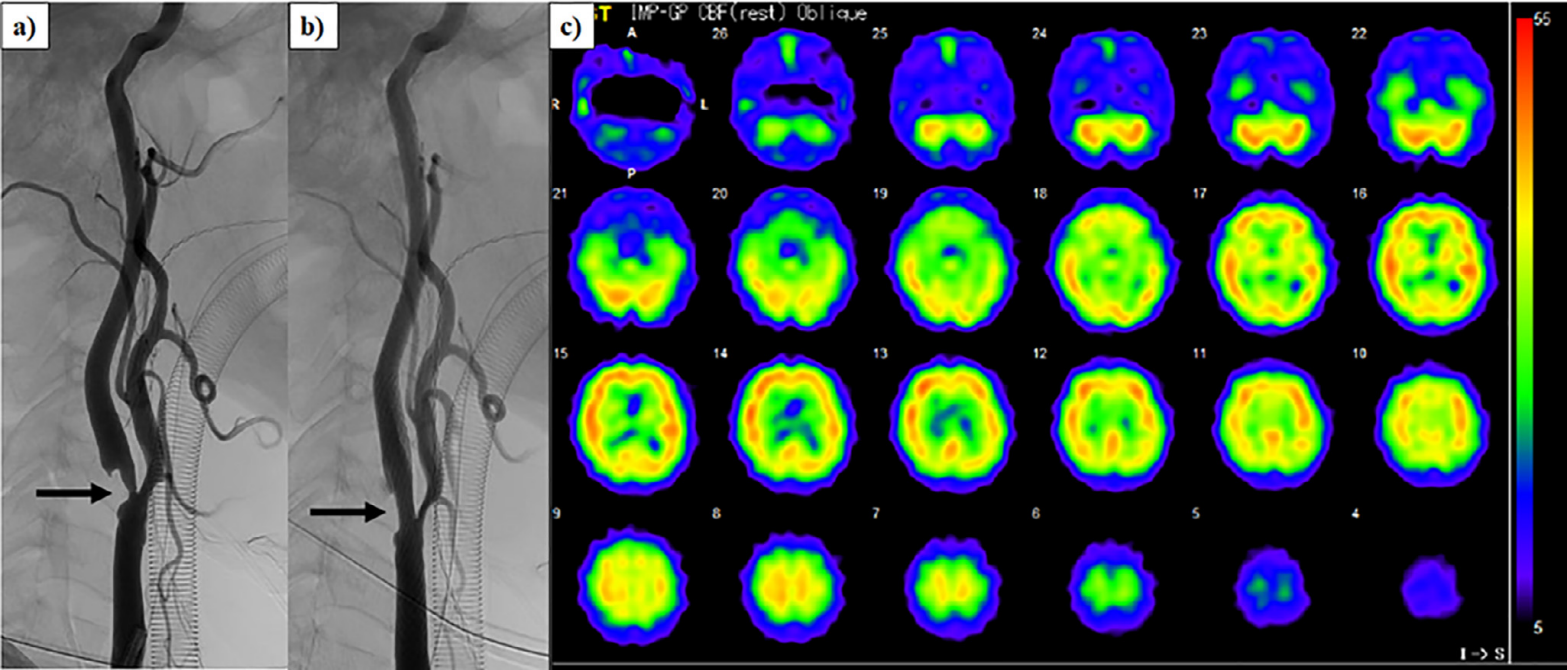
a) Severe stenosis of right internal carotid artery (arrow) demonstrated by cerebral angiography. b) Improvement of stenotic lesion of the right internal carotid artery (arrow) after carotid artery stenting. c)^123^I-N-isopropyl-p-iodoamphetamine single-photon emission computed tomography demonstrating no decreases in right cerebral blood flow.

**Fig. 2:**
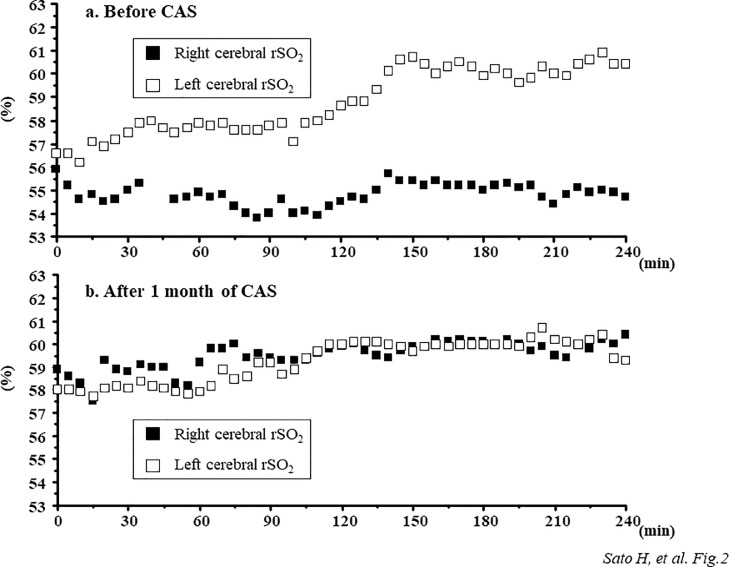
Changes in cerebral rSO_2_ during HD before and after CAS procedure. a) Right cerebral rSO_2_ (closed square) gradually increased from the initiation to the end of HD, whereas left cerebral rSO_2_ (open square) was maintained during HD before CAS. b) Differences in changes in cerebral rSO_2_ between bilateral side disappeared after CAS. CAS, carotid artery stenting; HD, hemodialysis; rSO_2_, regional oxygen saturation.

Our patient was a 68-year-old man with diabetes mellitus, hypertension, and ischemic heart disease, who underwent HD therapy for the treatment of end-stage renal failure for 6 years. Five years prior to CAS, a neck ultrasound was performed to screen for carotid atherosclerosis and revealed an asymptomatic right internal carotid artery stenosis. The patient's right internal carotid artery stenosis was 54%, based on the North American Symptomatic Carotid Endarterectomy Trial (NASCET) criteria, which corresponded to an intermediate stenosis. In contrast, no left carotid artery stenosis was revealed and there were no abnormalities on his neurological examinations. Thereafter, the right internal carotid stenosis was confirmed to gradually develop by neck ultrasound evaluation. One month prior, the degree of his right internal carotid stenosis and its influence on cerebral circulation were evaluated by the cerebral angiography and ^123^I-N-isopropyl-p-iodoamphetamine (IMP) single-photon emission computed tomography (SPECT). Cerebral angiography showed severe stenosis in the right internal carotid artery of 74% based on NASCET measurements (Fig. 1a), whereas no decreases in the right cerebral blood flow were confirmed using ^123^I-IMP SPECT (Fig. 1c). In this case, CAS was successfully performed without complications because of the progression of the right internal carotid artery stenosis (Fig. 1b). To evaluate the influence of the right internal carotid artery stenosis on intradialytic cerebral circulation and oxygenation, cerebral regional oxygen saturation (rSO_2_) at bilateral forehead was measured using the INVOS 5100c oxygen saturation monitor (Covidien Japan, Japan) during HD before and after 1 month of the CAS procedure. In each HD session before and after CAS, there were little differences in changes in the body weight (from 51.5 to 48.7 kg vs from 51.7 to 48.6 kg), systemic blood pressure (from 167/79 to 162/91 mmHg vs from 173/81 to 164/78 mmHg), and relative changes in blood volume (−8.4% vs −10.2%). Before CAS, pre-HD cerebral rSO_2_ values at bilateral sides were almost similar (right side; 56.6%, left side; 55.9%). Right cerebral rSO_2_ was maintained during HD, whereas left cerebral rSO_2_ gradually increased to 60.4% after HD (Fig. 2a). However, the differences in intradialytic cerebral rSO_2_ changes between bilateral sides disappeared after CAS (Fig. 2b).

## Discussion

In patients undergoing HD, the prevalence of carotid artery stenosis is approximately 6% to 10%, which is 5- to 10- fold higher than that of the general population [2], [3], [4]. Regarding the efficacy of carotid revascularization in stenosis, the threshold for achieving surgical benefit in asymptomatic patients with at least a 3-year life expectancy and stenosis >60% is recommended not to exceed 3% in the perioperative stroke and death rate [6,9]. In 2016, after the CAS procedure for HD patients with symptomatic and asymptomatic carotid artery stenosis, the 30-day perioperative stroke and death rate were 5.5% and 3.1%, respectively; therefore, avoiding CAS in HD patients was recommended [10]. However, new clinical findings reported that the 30-day carotid revascularization outcomes using the CAS procedure for HD patients with an asymptomatic carotid stenosis led to satisfying results (stroke, 0.9%; death rate, 1.8%), in addition to the results of no significant differences in these outcomes between dialysis and non-dialysis patients [6]. Therefore, carotid revascularization, including CAS, would be considered appropriate [5,6]. In our case, the right internal carotid artery stenosis reached 74%, although the patient was asymptomatic; therefore, CAS was performed in the right internal carotid lesion without perioperative complications.

In the present case, there were remarkable differences in intradialytic changes in cerebral rSO_2_ between the ipsilateral and contralateral side before CAS. These differences might be explained by the reason that collateral compensation of blood flow from the contralateral side through the circle of Willis mitigates the impairment in the ipsilateral cerebral circulation [11]. With brain blood flow computed simulation in the presence of internal carotid artery stenosis (67%) and a collateralized circle of Willis, the peak systolic, end diastolic, and mean flow at a contralateral internal carotid artery increased in contrast to the decrease in flow at an ipsilateral internal carotid artery [12]. Therefore, cerebral blood flow at a contralateral side might surge in patients with carotid artery stenosis to compensate for the decrease in an ipsilateral cerebral blood flow and perfusion pressure. Additionally, in the clinical setting of HD therapy, ultrafiltration is beneficial for the adjustment of the body-fluid excess; however it sometimes leads to a decrease in the blood pressure and relative blood volume changes during HD. A patient undergoing HD was previously reported to suffer from recurrent reversible neurological deficits occurring only during HD due to severe left carotid artery stenosis [13]. Therefore, cerebral circulation is possible to receive a negative impact of ultrafiltration-associated hemodynamic stress during HD, which would lead to the intradialytic differences between the ipsilateral and contralateral cerebral oxygenation in HD patients with carotid artery stenosis. Furthermore, in this case, the differences in intradialytic cerebral oxygenation between bilateral sides disappeared after CAS. This finding may imply that no deterioration of the right cerebral blood flow due to the improvement in right cerebral circulation after CAS may have occurred even under the ultrafiltration. Regarding the usefulness of cerebral oxygenation monitoring using NIRS in patients with carotid artery stenosis, changes in cerebral rSO_2_ were reportedly beneficial to detect ischemic intolerance during CAS [14] and predict the cerebral hyperperfusion syndrome after CAS [14,15]. Based on the present case, intradialytic cerebral oxygenation monitoring might disclose intradialytic changes in the cerebral blood flow distribution between the ipsilateral and contralateral side in HD patients with carotid artery stenosis. However, the association between changes in cerebral blood flow and those in cerebral oxygenation during HD with ultrafiltration could not be directly observed and remains uncertain; therefore, further studies are needed to clarify their relationship.

In conclusion, we report a HD case with the differences in intradialytic cerebral oxygenation changes between the ipsilateral and contralateral side of internal carotid artery stenosis and with the disappearance of these differences after CAS. Our experience demonstrates that changes in cerebral oxygenation during HD might disclose intradialytic changes in the cerebral blood flow distribution between the ipsilateral and contralateral side in HD patients with carotid artery stenosis.

## Statement of Ethics

Informed consent was obtained from the patient whose case is described in the manuscript. The measurement of cerebral oxygenation was approved by the Institutional Review Board of our hospital and conformed to the provisions of the Declaration of Helsinki (as revised in Tokyo in 2004).

## Authors’ Contributions

All authors contributed to the writing and intellectual content of the manuscript.
